# International estimated fetal weight standards of the INTERGROWTH‐21^st^ Project

**DOI:** 10.1002/uog.17347

**Published:** 2017-03-05

**Authors:** J. Stirnemann, J. Villar, L. J. Salomon, E. Ohuma, P. Ruyan, D. G. Altman, F. Nosten, R. Craik, S. Munim, L. Cheikh Ismail, F. C. Barros, A. Lambert, S. Norris, M. Carvalho, Y. A. Jaffer, J. A. Noble, E. Bertino, M. G. Gravett, M. Purwar, C. G. Victora, R. Uauy, Z. Bhutta, S. Kennedy, A. T. Papageorghiou, for the International Fetal and Newborn Growth Consortium for the 21st Century (INTERGROWTH‐21st), Scientific Advisory Committee, M Katz, MK Bhan, C Garza, S Zaidi, A Langer, PM Rothwell, Sir D Weatherall, Steering Committees, INTERGROWTH‐21st, ZA Bhutta, J Villar, S Kennedy, DG Altman, FC Barros, E Bertino, F Burton, M Carvalho, L Cheikh Ismail, WC Chumlea, MG Gravett, YA Jaffer, A Lambert, P Lumbiganon, JA Noble, RY Pang, AT Papageorghiou, M Purwar, J Rivera, C Victora, INTERBIO‐21st, R Uauy, S Kennedy, J Villar, DG Altman, FC Barros, J Berkley, F Burton, M Carvalho, L Cheikh Ismail, WC Chumlea, A Lambert, S Munim, S Norris, F Nosten, AT Papageorghiou, C Victora, Executive Committee, J Villar, DG Altman, L Cheikh Ismail, S Kennedy, A Lambert, JA Noble, AT Papageorghiou, ZA Bhutta, In addition for INTERBIO 21st, R Craik, R Uauy, Project Coordinating Unit, J Villar, L Cheikh Ismail, S Kennedy, A Lambert, AT Papageorghiou, M Shorten, L Hoch, HE Knight, EO Ohuma, C Cosgrove, I Blakey, S Ash, R Craik, Data Analysis Group, DG Altman, EO Ohuma, E Staines Urias, J Villar, Data Management Group, DG Altman, F Roseman, N Kunnawar, SH Gu, JH Wang, MH Wu, M Domingues, P Gilli, L Juodvirsiene, L Hoch, N Musee, H Al‐Jabri, S Waller, C Cosgrove, D Muninzwa, EO Ohuma, D Yellappan, A Carter, D Reade, R Miller, In addition for INTERBIO 21st, I Ahmed, S Ash, C Condon, M Mainwaring, D Muninzwa, MF da Silveira, E Staines Urias, L Walusuna, S Wiladphaingern, Ultrasound Group, AT Papageorghiou, L Salomon, A Leston, A Mitidieri, F Al‐Aamri, W Paulsene, J Sande, WKS Al‐Zadjali, C Batiuk, S Bornemeier, M Carvalho, M Dighe, P Gaglioti, N Jacinta, S Jaiswal, JA Noble, K Oas, M Oberto, E Olearo, MG Owende, J Shah, S Sohoni, T Todros, M Venkataraman, S Vinayak, L Wang, D Wilson, QQ Wu, S Zaidi, Y Zhang, P Chamberlain, D Danelon, I Sarris, J Dhami, C Ioannou, CL Knight, R Napolitano, S Wanyonyi, C Pace, V Mkrtychyan, In addition for INTERBIO‐21st:, M Buckle, N Jackson, A Mitidieri, S Munim, H Mwangudzah, T Norris, J Shah, G Zainab, Anthropometry Group, L Cheikh Ismail, WC Chumlea, F Al‐Habsi, ZA Bhutta, A Carter, M Alija, JM Jimenez‐Bustos, J Kizidio, F Puglia, N Kunnawar, H Liu, S Lloyd, D Mota, R Ochieng, C Rossi, M Sanchez Luna, YJ Shen, HE Knight, DA Rocco, IO Frederick, In addition for INTERBIO‐21st:, J Kizidio, B Monyepote, M Salim, R Salam, VI Carrara, Laboratory Processing Group, R Craik, D Alam, Y Guman, J Kilonzo, A Min, V Ngami, I Olivera, G Deutsch, Neonatal Group, ZA Bhutta, E Albernaz, M Batra, BA Bhat, E Bertino, P Di Nicola, F Giuliani, I Rovelli, K McCormick, R Ochieng, RY Pang, V Paul, V Rajan, A Wilkinson, R Uauy, A Varalda, Environmental Health Group, B Eskenazi, A Bradman, O Burnham, LA Corra, H Dolk, F Farhi, D Finkton, J Golding, A Matijasevich, T de Wet, J Villar, JJ Zhang, Neurodevelopment Group, A Stein, M Fernandes, A Abubakar, J Acedo, L Aranzeta, L Cheikh Ismail, F Giuliani, D Ibanez, S Kennedy, M Kihara, E de Leon, CR Newton, S Savini, A Soria‐Frisch, J Villar, K Wulff, Participating countries and local investigators, FC Barros, M Domingues, S Fonseca, A Leston, A Mitidieri, D Mota, IK Sclowitz, MF da Silveira, RY Pang, YP He, Y Pan, YJ Shen, MH Wu, QQ Wu, JH Wang, Y Yuan, Y Zhang, M Purwar, A Choudhary, S Choudhary, S Deshmukh, D Dongaonkar, M Ketkar, V Khedikar, N Kunnawar, C Mahorkar, I Mulik, K Saboo, C Shembekar, A Singh, V Taori, K Tayade, A Somani, E Bertino, P Di Nicola, M Frigerio, G Gilli, P Gilli, M Giolito, F Giuliani, M Oberto, L Occhi, C Rossi, I Rovelli, F Signorile, T Todros, W Stones, M Carvalho, J Kizidio, R Ochieng, J Shah, S Vinayak, N Musee, C Kisiang'ani, D Muninzwa, In addition for INTERBIO‐21st:, J Kilonzo, J Sande, J Berkley, B Kemp, H Barsosio, S Mwakio, H Mwangudzah, V Ngami, M Salim, A Seale, L Walusuna, YA Jaffer, J Al‐Abri, J Al‐Abduwani, FM Al‐Habsi, H Al‐Lawatiya, B Al‐Rashidiya, WKS Al‐Zadjali, FR Juangco, M Venkataraman, H Al‐Jabri, D Yellappan, S Munim, G Zainab, I Ahmed, D Alam, A Raza, R Salam, S Norris, Y Guman, T Lephoto, S Macauley, L Malgas, F Nosten, N Jackson, R McGready, A Min, VI Cararra, S Wiladphaingern, S Kennedy, L Cheikh Ismail, A Lambert, S Lloyd, R Napolitano, EO Ohuma, AT Papageorghiou, B Patel, F Puglia, F Roseman, S Roseman, C Ioannou, I Sarris, In addition for INTERBIO‐21st:, S Ash, M Baricco, A Capp, R Craik, S Hussein, A Laister, T Lewis, E Maggiora, T Norris, M Sharps, A Varalda, R Carew, MG Gravett, C Batiuk, M Batra, S Bornemeier, M Dighe, K Oas, W Paulsene, D Wilson, IO Frederick, HF Andersen, SE Abbott, AA Carter, H Algren, DA Rocco, TK Sorensen, D Enquobahrie, S Waller

**Affiliations:** ^1^ Maternité Necker‐Enfants Malades AP‐HP & EA7328 Université Paris Descartes Paris France; ^2^ Nuffield Department of Obstetrics & Gynaecology and Oxford Maternal & Perinatal Health Institute, Green Templeton College University of Oxford Oxford UK; ^3^ Collège Français d'Echographie Foetale – CFEF France; ^4^ Centre for Statistics in Medicine, Nuffield Department of Orthopaedics, Rheumatology & Musculoskeletal Sciences University of Oxford Oxford UK; ^5^ School of Public Health Peking University Beijing China; ^6^ Shoklo Malaria Research Unit Maesod Tak Thailand; ^7^ Division of Women & Child Health The Aga Khan University Karachi Pakistan; ^8^ Programa de Pós‐Graduação em Saúde e Comportamento Universidade Católica de Pelotas Pelotas RS Brazil; ^9^ Programa de Pós‐Graduação em Epidemiologia Universidade Federal de Pelotas Pelotas RS Brazil; ^10^ Developmental Pathways For Health Research Unit, Department of Paediatrics & Child Health University of the Witwatersrand Johannesburg South Africa; ^11^ Faculty of Health Sciences Aga Khan University Nairobi Kenya; ^12^ Department of Family & Community Health, Ministry of Health Muscat Sultanate of Oman; ^13^ Department of Engineering Science University of Oxford Oxford UK; ^14^ Dipartimento di Scienze Pediatriche e dell'Adolescenza, Cattedra di Neonatologia Università degli Studi di Torino Torino Italy; ^15^ Global Alliance to Prevent Prematurity and Stillbirth (GAPPS) Seattle WA USA; ^16^ Nagpur INTERGROWTH‐21^st^ Research Centre Ketkar Hospital Nagpur India; ^17^ Division of Paediatrics Pontifical Universidad Catolica de Chile Chile; ^18^ London School of Hygiene and Tropical Medicine London UK; ^19^ Center for Global Child Health Hospital for Sick Children Toronto ON Canada

**Keywords:** birth weight, fetal growth, gestational age, screening, ultrasound

## Abstract

**Objective:**

Estimated fetal weight (EFW) and fetal biometry are complementary measures used to screen for fetal growth disturbances. Our aim was to provide international EFW standards to complement the INTERGROWTH‐21^st^ Fetal Growth Standards that are available for use worldwide.

**Methods:**

Women with an accurate gestational‐age assessment, who were enrolled in the prospective, international, multicenter, population‐based Fetal Growth Longitudinal Study (FGLS) and INTERBIO‐21^st^ Fetal Study (FS), two components of the INTERGROWTH‐21^st^ Project, had ultrasound scans every 5 weeks from 9–14 weeks' until 40 weeks' gestation. At each visit, measurements of fetal head circumference (HC), biparietal diameter, occipitofrontal diameter, abdominal circumference (AC) and femur length (FL) were obtained blindly by dedicated research sonographers using standardized methods and identical ultrasound machines. Birth weight was measured within 12 h of delivery by dedicated research anthropometrists using standardized methods and identical electronic scales. Live babies without any congenital abnormality, who were born within 14 days of the last ultrasound scan, were selected for inclusion. As most births occurred at around 40 weeks' gestation, we constructed a bootstrap model selection and estimation procedure based on resampling of the complete dataset under an approximately uniform distribution of birth weight, thus enriching the sample size at extremes of fetal sizes, to achieve consistent estimates across the full range of fetal weight. We constructed reference centiles using second‐degree fractional polynomial models.

**Results:**

Of the overall population, 2404 babies were born within 14 days of the last ultrasound scan. Mean time between the last scan and birth was 7.7 (range, 0–14) days and was uniformly distributed. Birth weight was best estimated as a function of AC and HC (without FL) as log(EFW) = 5.084820 − 54.06633 × (AC/100)^3^ − 95.80076 × (AC/100)^3^ × log(AC/100) + 3.136370 × (HC/100), where EFW is in g and AC and HC are in cm. All other measures, gestational age, symphysis–fundus height, amniotic fluid indices and interactions between biometric measures and gestational age, were not retained in the selection process because they did not improve the prediction of EFW. Applying the formula to FGLS biometric data (*n* = 4231) enabled gestational age‐specific EFW tables to be constructed. At term, the EFW centiles matched those of the INTERGROWTH‐21^st^ Newborn Size Standards but, at < 37 weeks' gestation, the EFW centiles were, as expected, higher than those of babies born preterm. Comparing EFW cross‐sectional values with the INTERGROWTH‐21^st^ Preterm Postnatal Growth Standards confirmed that preterm postnatal growth is a different biological process from intrauterine growth.

**Conclusions:**

We provide an assessment of EFW, as an adjunct to routine ultrasound biometry, from 22 to 40 weeks' gestation. However, we strongly encourage clinicians to evaluate fetal growth using separate biometric measures such as HC and AC, as well as EFW, to avoid the minimalist approach of focusing on a single value. © 2016 Authors. Ultrasound in Obstetrics & Gynecology published by John Wiley & Sons Ltd on behalf of International Society of Ultrasound in Obstetrics and Gynecology.

## INTRODUCTION

One of the main objectives of antenatal care is screening for fetal growth disturbances^1^. Although biomarkers in maternal blood have shown some potential for detecting fetal growth restriction^2^, ^3^, a recent systematic review suggested that none is sufficiently accurate to be recommended for use in clinical practice^4^. Clinicians, therefore, still rely routinely on clinical markers, including ultrasound measurements, to identify fetuses at risk^5^.

Ultrasound evaluation of the fetus involves measuring head circumference (HC), abdominal circumference (AC) and femur length (FL), and the values can be combined to calculate an estimated fetal weight (EFW); this estimate is often used alone in clinical practice without considering the individual measurements. However, we believe that arguments concerning the most appropriate single parameter to use are inappropriate because clinicians should use all the tools available in their armamentarium for making crucial clinical decisions that have major implications for both mothers and newborns.

The development of international EFW standards is overdue, and these should share the same conceptual basis as the published INTERGROWTH‐21^st^ standards for HC, AC and FL, size at birth and postnatal growth in preterm infants^6^, ^7^, ^8^, ^9^. These standards would perfectly complement the World Health Organization (WHO) Child Growth Standards^10^, thereby enabling continuity of assessment of human growth from early pregnancy to childhood^11^. Therefore, the objectives of this component of the INTERGROWTH‐21^st^ Project were: (1) to develop a formula to estimate fetal weight based on ultrasound biometry and birth weight; and (2) to construct international EFW standards for fetuses at 22 to 40 weeks' gestation.

## SUBJECTS AND METHODS

INTERGROWTH‐21^st^ is an international, multicenter, population‐based project consisting of a number of components, including the Fetal Growth Longitudinal Study (FGLS) and INTERBIO‐21^st^ Fetal Study (FS).

FGLS was conducted between 27 April 2009 and 2 March 2014 in eight urban areas: the cities of Pelotas (Brazil), Turin (Italy), Muscat (Oman), Oxford (UK) and Seattle (USA); the Shunyi County of Beijing (China); the central area of Nagpur (India); and the Parklands suburb of Nairobi (Kenya). The primary aim was to study longitudinally the health and development of fetuses into infancy, by monitoring growth, health, nutrition and neurodevelopment from less than 14 weeks' gestation to 2 years of age, so as to produce prescriptive growth standards to complement the existing WHO Child Growth Standards. This was achieved by studying a cohort of healthy, well‐nourished, pregnant women who were at low risk of adverse maternal and perinatal outcomes at both population and individual levels. The study details have been described elsewhere^9^, ^12^.

In contrast, FS recruited an unselected cohort of pregnant women, between 8 February 2012 and 24 December 2015, from three FGLS sites (Pelotas, Nairobi, Oxford), and three new sites (Aga Khan University Hospital, Karachi, Pakistan; Shoklo Malaria Research Unit, Mae Sot, Thailand; and Baragwanath Hospital, Soweto, South Africa). The primary aim was to study the effects of various intrauterine exposures (e.g. malnutrition, anemia, human immunodeficiency virus, malaria) on growth, health, nutrition, neurodevelopment and the epigenome, over the same developmental age range, i.e. from less than 14 weeks' gestation to 2 years of age.

To develop the EFW formula requires as many pregnancies as possible that have a standardized scan and birth‐weight measurement. In order to achieve this we included fetuses from both FGLS and FS; only those that had an ultrasound scan within 14 days of birth were included in the calculations. To develop the international standards for EFW, the formula derived was then applied to the healthy FGLS population from which the International Fetal Growth Standards were produced^6^.

The INTERGROWTH‐21^st^ Project was approved by the Oxfordshire Research Ethics Committee ‘C’ (reference: 08/H0606/139), the research ethics committees of the individual participating institutions and the corresponding regional health authorities in which the project was implemented. Participants provided written consent to be involved in the study.

### Standard procedures

In both studies women were recruited at less than 14 weeks' gestation. All women underwent ultrasound measurement of fetal crown–rump length (CRL) using standardized methodology^13^, ^14^. In FGLS, gestational age was based on the date of the last menstrual period (LMP) provided it was certain, the woman had a regular 24–32‐day menstrual cycle and she had not been using hormonal contraception or breastfeeding in the preceding 2 months, and any discrepancy between the gestational ages based on LMP and CRL, between 9 + 0 and 13 + 6 weeks, was ≤ 7 days. In FS, gestational age was determined by CRL measurement alone, using the same formula loaded onto all study ultrasound machines^15^; if known, the date of the LMP was recorded.

Following the dating scan, women were scanned every 5 weeks (±1 week), so that the possible ranges were 14–18, 19–23, 24–28, 29–33, 34–38 and 39–42 weeks' gestation. At each visit, fetal HC, biparietal diameter (BPD), occipitofrontal diameter (OFD), AC and FL were measured three times from three separately obtained ultrasound images of each structure. The detailed measurement protocols, including graphical displays of measurement techniques, and the unique standardization procedures for all measurements and sonographer training have been reported elsewhere^13^, ^16^. In addition, all documentation, protocols, quality‐control procedures, data collection forms and electronic transfer strategies are freely available on the INTERGROWTH‐21^st^ website.

Briefly, head measurements were taken in an axial view at the level of the thalami, with an angle of insonation as close as possible to 90°. The head had to be oval in shape, symmetrical, centrally positioned and filling at least 30% of the monitor screen. The midline echo (representing the falx cerebri) had to be broken anteriorly, at a third of its length, by the cavum septi pellucidi. The thalami had to be located symmetrically on either side of the midline. Calipers were then placed on the outer border of the parietal bones (outer to outer) at the widest or longest part of the skull for the BPD and OFD, respectively; HC was measured using the ellipse facility on the outer border of the skull.

AC measurements were taken in a cross‐sectional view of the fetal abdomen as close as possible to circular in shape, with the umbilical vein in the anterior third (at the level of the portal sinus), with the stomach bubble visible. The sonographer was instructed to avoid applying too much pressure with the transducer, which can distort the circular shape of the fetal abdomen. The abdomen had to fill at least 30% of the monitor screen, and the spine had to be at either the 3 or 9 o'clock position to avoid internal shadowing; the kidneys and bladder had not to be visible. For the measurements, the contour of the ellipse was placed on the outer border of the abdomen.

Finally, FL was measured using a longitudinal view of the fetal thigh closest to the probe and with the femur as close as possible to the horizontal plane. The angle of insonation of the ultrasound beam was about 90°, with the full length of the bone visualized, unobscured by shadowing from adjacent bony parts, and the femur had to fill at least 30% of the monitor screen. The intersection of the calipers was placed on the outer borders of the edges of the femoral diaphysis (outer to outer) ensuring clear femoral edges; ultrasound artifacts of the femoral edges such as the proximal trochanter or pointed femoral spurs were not included in the measurement (detailed methods and a graphical display of how the bone structures are localized are available on the INTERGROWTH‐21^st^ website).

The same type of ultrasound machine, a Philips HD‐9 with curvilinear abdominal transducers C5‐2, C6‐3 and V7‐3 (Philips Ultrasound, Bothell, WA, USA), was used at all sites. To avoid expected value bias, the machine was adapted so that fetal measurements were not visible to the sonographer on the screen. Only after three measurements of each structure had been recorded were the average values revealed for clinical purposes. All ultrasound data were submitted electronically to the study database. Data were entered locally directly onto the web‐based system^17^.

After taking each set of measurements, sonographers scored the quality of their images on the basis of standard image‐scoring criteria^18^, ^19^. Images that did not score the maximum number of points were repeated until the best possible score was achieved. The quality‐control methods used across all sites are described in detail elsewhere^18^, ^20^.

Birth weight was measured within 12 h of birth using identical electronic scales (Seca, Hangzhou, China) at all sites. The equipment, which was calibrated twice a week, was selected for accuracy, precision and robustness, as shown previously^21^. Measurement procedures were standardized on the basis of WHO recommendations to ensure maximum validity and each measurement was collected independently by two study anthropometrists^22^, ^23^. If the difference between the two measurements exceeded the maximum allowable difference of 5 g, then both observers independently retook that measurement a second and, if necessary, a third time. The training, standardization, monitoring processes and quality‐control methods used across all sites are described in detail elsewhere^22^, ^23^.

### Statistical analysis

#### Estimation of fetal weight

From the FGLS and FS cohorts, we identified all live babies without any congenital abnormality who were born at > 24 weeks' gestation and within 14 days of the last ultrasound scan. Given the study design, we expected the births to have occurred uniformly between 0 and 14 days after the last ultrasound scan, i.e. we expected there to be a mean time of 7–8 days between the last scan and birth. This cut‐off allowed a greater number of births at low gestational ages to be included, for which most of the existing formulae have been prone to prediction error, probably because scant data exist for estimation^24^. Potential predictors for birth weight were:

- HC, BPD, OFD, AC and FL, in cm or transformed into *Z*‐scores using the INTERGROWTH‐21^st^ equations^6^;
- gestational age on the day of the last scan, in weeks;
- symphysis–fundus height, in cm;
- amniotic fluid, assessed by the deepest vertical pool and amniotic fluid index in cm;
- cross‐sectional head area and abdominal area computed from their orthogonal diameters, in cm^2^.

We hypothesized that the contribution of any given anthropometric measurement to EFW might vary with gestational age. Therefore, we also considered interactions between HC, BPD, OFD, AC and FL and gestational age on the day of the last scan. Statistical modeling was conducted using second‐degree fractional polynomials^25^.

Some prediction bias would be expected because of significant growth between the day of the last scan and birth^24^, ^26^, ^27^. We addressed this issue by calculating the expected EFW on the day of the ultrasound scan, using the following steps: (1) in pregnancies from FGLS and FS delivering within 14 days from the last scan, we developed a model to predict birth weight from the most recent ultrasound measurements; (2) in the complete FGLS dataset, we calculated EFW from ultrasound biometry using the previous model and fitted a second‐degree fractional polynomial for mean weight as a function of gestational age between 22 and 40 weeks; (3) returning to the dataset of births within 14 days (step 1), we calculated, for each fetus, the expected weight at the time of the last scan by subtracting the average weight gain between the time of the last scan and birth using the model built in step 2; (4) this calculated weight was then used for further modeling.

As expected, owing to the prospective, population‐based design of FGLS, most births occurred close to 40 weeks' gestation, meaning that the scatter of observations across the 22–40‐week window was very uneven. We were aware that estimation using the complete dataset would yield very accurate estimates at 40 weeks' gestation, where the greatest contribution of the data is found, but with limited model validity for lower birth weights. To overcome this problem and allow accurate birth‐weight estimation over the whole range of observed data, we constructed a bootstrap model selection and estimation procedure based on resampling of the complete dataset under an approximately uniform distribution of birth weight^28^, ^29^, ^30^, i.e. birth weight was divided into 500‐g strata and each sample was built by randomly selecting five observations with replacement from each stratum. In a first resampling run of 100 samples, candidate models, which include three elements (the variables, the coefficients and the respective fractional polynomial powers), were elicited using the backward elimination algorithm described by Ambler and Royston^31^, which provides protection against over‐fitting. In a second step, the coefficients of all candidate models were estimated in B = 1000 bootstrap samples: in each sample, a single model was selected using Akaike's Information Criterion. Candidate models were then ordered by their frequency of selection within the 1000 samples, and the five most frequent models were kept for further assessment of goodness of fit.

Assessment of goodness of fit in the complete dataset relied on inspection of residuals with quantile–quantile (q‐q) plots and residuals *vs* fitted plots. Given that we estimated fetal weight at the time of the last scan using an average model for growth, we investigated the bias of our model for EFW by calculating the mean of percent prediction errors defined by the formula (100 × (EFW − birth weight)/birth weight), for decreasing time‐to‐birth intervals (i.e. from 14 to 0 days). Finally, we also calculated the absolute percent prediction error defined by the mean of the absolute prediction errors.

#### Construction of reference centiles

The construction of reference centiles was based solely on FGLS data. The sample size was based on pragmatic and statistical considerations; the latter focused on the precision and accuracy of one extreme centile, i.e. the 3^rd^ or 97^th^ centile, and regression‐based reference limits^32^, ^33^. We have shown that a sample of 4000 women would obtain a precision of 0.03 SD at the 3^rd^ or 97^th^ centile. Further details on the precision obtained at the 5^th^ or 10^th^ centile by sample size (ranging from 500–6000) have been included in a table in a previous publication^34^.

The data from all the study sites were pooled to construct the Fetal Growth Standards^6^, ^12^, using the same statistical approach adopted by WHO in constructing their Child Growth Standards^10^. The statistical methods used were based on published recommendations complemented by recent scientific reviews^5^, ^35^, ^36^, ^37^, ^38^. Our overall aim was to produce centiles that change smoothly with age and maximize simplicity without compromising model fit.

We explored the following statistical methods: mean and SD method using fractional polynomials^25^; Cole's lambda (λ), mu (μ), and sigma (σ) (LMS) method^39^, ^40^, ^41^, which estimates three age‐specific parameters (the median (μ), coefficient of variation (σ), and a Box–Cox power transformation at each gestational age to remove skewness (λ), thereby making the data roughly normally distributed); the LMST method (λ, μ, σ, assuming Box–Cox t distribution), which assumes a shifted and scaled (truncated) t distribution to take account of skewness and leptokurtosis^42^; and the LMSP method (λ, μ, σ, assuming Box–Cox power exponential distribution), which assumes a Box–Cox power exponential distribution to take account of skewness, platykurtosis and leptokurtosis^43^. Furthermore, to present the curves, we assessed three smoothing techniques: fractional polynomials, cubic splines and penalized splines^25^, ^44^, ^45^.

Using de‐trended q‐q plots (worm plots), significant evidence of deviations from normality was seen so we resorted to using the more complex LMS, LMST and LMSP methods allowing for skewness and kurtosis^46^.

As most of the women had four to six ultrasound scans, the effect of correlated data within fetuses was investigated. First, in a sensitivity analysis, a random observation time was sampled for each fetus and the modeled centiles in this subset were compared visually with the complete dataset. The approach is justified by the experimental design of the study, which ensures non‐informative observation times^47^. This analysis showed minimal or no change in estimated centiles (median, 3^rd^ and 97^th^ centiles) over the whole 22–40 weeks' gestational‐age range. Second, we considered mixed‐effect models accounting for repeated measurements within LMS, LMST and LMSP frameworks. This analysis also showed no impact on the estimated centiles. The best fit was found using a three‐parameter Box–Cox Gaussian distribution (i.e. the LMS method) for the response variable with a second‐degree fractional polynomial functional form for gestational age. This method also gives estimated SDs of EFW, allowing estimation of centiles.

Goodness of fit for the overall model was assessed by comparing empirical centiles (calculated per completed week of gestation) with the fitted centiles, using de‐trended q‐q plots of the residuals across gestational age^46^, and plots of residuals *vs* fitted values.

All analyses were carried out in R statistical software^48^ using the Generalised Additive Models for Location, Scale and Shape (GAMLSS) framework^49^, ^50^.

## RESULTS

To create an EFW formula, the subsets of 2404 babies in the FGLS (*n* = 1556) and FS (*n* = 848) who were born within 14 days of the last ultrasound scan were examined (Table 1): 130 (5.4%) were born preterm (< 37 weeks' gestation) and 78 (3.2%) were born term with low birth weight (< 2500 g and ≥ 37 weeks' gestation). The mean time between the last ultrasound scan and birth was 7.7 (range, 0–14) days and was uniformly distributed, except for 0 days (i.e. birth on day of last scan), which occurred in only 34 (1.4%) cases.

**Table 1:** Gestational age at birth and birth weight of a subset of babies in the INTERBIO‐21^st^ Fetal Study (FS) and the Fetal Growth Longitudinal Study (FGLS) of the INTERGROWTH‐21^st^ Project who were born within 14 days of last ultrasound scan

Parameter	FS (*n* = 848)	FGLS (*n* = 1556)	Total (*n* = 2404)
Gestational age at birth			
< 28 weeks	2 (0.2)	1 (0.1)	3 (0.1)
28–32 weeks	3 (0.4)	6 (0.4)	9 (0.4)
32–37 weeks	56 (6.6)	62 (4.0)	118 (4.9)
37–43 weeks	787 (92.8)	1487 (95.6)	2274 (94.6)
Birth weight			
< 1000 g	1 (0.1)	2 (0.1)	3 (0.1)
1000–1499 g	5 (0.6)	1 (0.1)	6 (0.2)
1500–1999 g	9 (1.1)	15 (1.0)	24 (1.0)
2000–2499 g	79 (9.3)	76 (4.9)	155 (6.4)
≥ 2500 g	754 (88.9)	1462 (94.0)	2216 (92.2)

Data are given as *n* (%).

Following correction for potential growth between the last scan and birth (steps 1–4 in the statistical methods), the actual fetal weight at the time of the last scan was best estimated as a function of AC and HC with the following formula:

$$\begin{array}{cc} \log\left(EFW\right) & =5.084820-54.06633\times {\left(\mathrm{AC}/100\right)}^{3}\\ & -95.80076\times {\left(\mathrm{AC}/100\right)}^{3}\times \log\left(\mathrm{AC}/100\right)\\ & +3.136370\times \left(HC/100\right) \end{array}$$

where EFW is expressed in g, AC and HC in cm, and the log function designates the natural logarithm.

None of the other covariates including FL, BPD, OFD, gestational age, symphysis–fundus height, amniotic fluid indices or interactions between biometric measurements and gestational age was retained in the selection process. This model suggests a linear relationship between log(EFW) and HC. Despite the negative coefficients, the two terms involving AC describe an increasing sigmoid‐shaped relationship between AC and birth weight (Figure S1) for a fixed HC value of 26 cm (the average value at 28 weeks' gestation^6^). The relationship between birth weight and HC is plotted in Figure S2, for a fixed AC value of 23 cm (the average at 28 weeks' gestation^6^).

The performance of the formula for EFW was assessed both by mean and absolute percent prediction errors; mean percent prediction error is used as a measure of potential bias of EFW due to growth between the last scan and birth, while mean absolute prediction error represents the dispersion of the errors. The mean percent prediction error steadily tended towards zero as the time interval between the last scan and birth decreased. Prediction error was −10.7% (95% CI, −12.1 to −9.4%) in babies born exactly 14 days after the last scan (*n* = 196) and −0.8% (95% CI, −2.3 to 0.6%) in those born within 1 day (*n* = 198) (Figure S3), showing that our model was unbiased for predicting weight at the time of the last scan and that the correction we applied to compensate for time to birth was appropriate. In the group born within 1 day of the last scan, the mean absolute prediction error was 7.6%, with 80%, 90% and 95% of predicted weights falling within 11%, 14% and 18% of the true birth weight, respectively.

**Figure 1:**
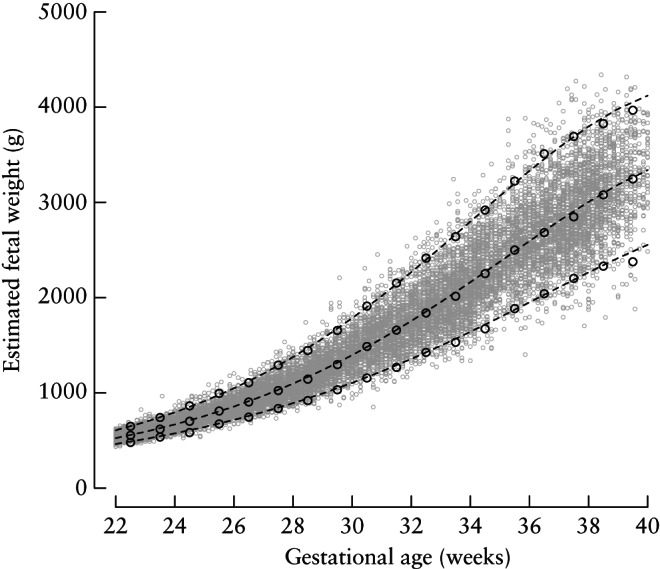
Empirical (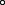) and smoothed (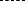) 3^rd^, 50^th^ and 97^th^ centiles for estimated fetal weight between 22 and 40 weeks' gestation.

Creation of the international EFW standards was based on the complete FGLS dataset. The gestational age‐specific observed and smoothed centiles for EFW are presented in Figure 1. Similarities between smoothed centile curves (3^rd^, 50^th^ and 97^th^ centiles) and observed values, assessed by gestational age‐specific comparisons, demonstrated excellent agreement. The overall differences between empirical and smoothed centiles were small, with mean ± SD differences of 16 ± 28 g, 13 ± 17 g and 5 ± 33 g for the 3^rd^, 50^th^ and 97^th^ centiles, respectively.

**Figure 2:**
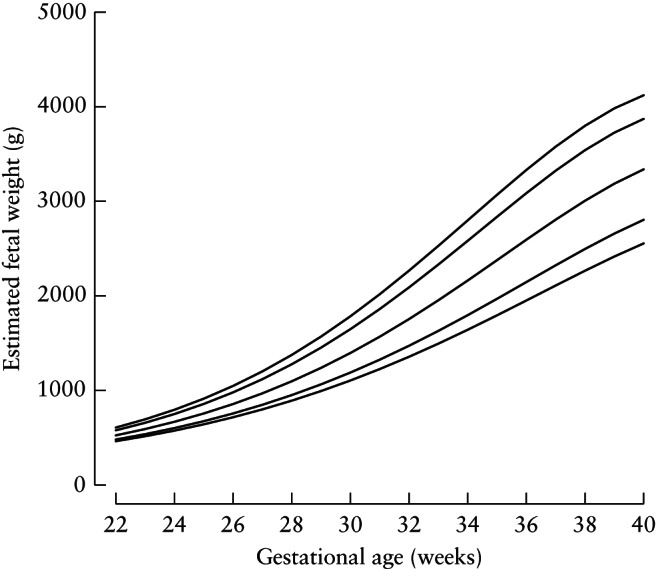
Smoothed 3^rd^, 10^th^, 50^th^, 90^th^ and 97^th^ centile curves for estimated fetal weight.

The 3^rd^, 10^th^, 50^th^, 90^th^ and 97^th^ fitted centile curves for EFW according to gestational age, which represent the international standards, are presented in Figure 2. The corresponding equations for λ(t), μ(t) and σ(t), are presented in Table 2, allowing readers to calculate *Z*‐scores. By estimating the EFW and knowing the gestational age, desired centiles can be calculated. For example, if AC = 26 cm and HC = 29 cm, at 30 + 0 weeks:

$$\begin{array}{cc} & \log\left(EFW\right)=5.084820-54.06633\times {\left(26/100\right)}^{3}\\ & -95.80076\times {\left(26/100\right)}^{3}\times \log\left(26/100\right)+3.136370\\ & \times \left(29/100\right)=7.312292\text{.} \end{array}$$

Therefore, EFW = exp(7.312292) = 1499 g.

**Table 2:** Equations for parameters and computation of *Z*‐scores and centiles for estimated fetal weight (EFW) in relation to gestational age (GA) in exact weeks

Parameter	Equation
Skewness	λ(GA) = − 4.257629 − 2162.234 × GA^− 2^ + 0.0002301829 × GA^3^
Mean	μ(GA) = 4.956737 + 0.0005019687 × GA^3^ − 0.0001227065 × GA^3^ × log(GA)
Coefficient of variation	σ(GA) = 10^− 4^ × (− 6.997171 + 0.057559 × GA^3^ − 0.01493946 × GA^3^ × log(GA))
*Z*‐score	Y = log(EFW)
	If λ(GA) = 0, Z(GA) = σ(GA)^− 1^ × log[Y/μ(GA)]
	If λ(GA) ≠ 0, Z(GA) = [σ(GA) × λ(GA)]^− 1^ × [(Y/μ(GA))^λ(GA)^ − 1]
Centiles	Z_α_defined by Pr(z ≤ Z_α_) = α for z ∼ N(0, 1), i. e. Z_α_ = Φ^− 1^(α)
	If λ(GA) = 0, log[C_α_(GA)] = μ(GA) × exp[σ(GA) × Z_α_]
	If λ(GA) ≠ 0, log[C_α_(GA)] = μ(GA) × [Z_α_ × σ(GA) × λ(GA) + 1]^1/λ(GA)^

To compute the corresponding *Z*‐score at 30 weeks' gestation, using the equations in Table 2 we must first calculate:

$$\begin{array}{cc} & \lambda \left(30\right)=–4.257629-2162.234\times 30^{–2}\\ & +0.0002301829\times 30^{3}=–0.4451729\text{.} \end{array}$$

$$\begin{array}{cc} & µ\left(30\right)=4.956737+0.0005019687\times 30^{3}\\ & –\;0.0001227065\times 30^{3}\times \;\log30=7.241468\text{.} \end{array}$$

$$\begin{array}{cc} & \sigma \left(30\right)=10^{–4}\times (–6.997171+0.057559\times 30^{3}\\ & –\;0.01493946\times 30^{3}\times \log30)=0.017517\text{.} \end{array}$$

Finally,

$$\begin{array}{cc} & Z={\left(0.017517\times –0.4451729\right)}^{–1}\times \\ & \;({\left(7.312292/7.241468\right)}^{–0.4451729}\;–\;1)=0.5617023\text{.} \end{array}$$

Similarly, the 3^rd^ centile (α = 0.03), i.e. *Z* = −1.88 at 30 + 0 weeks, is calculated as follows using the equations in Table 2:


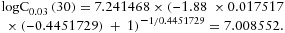


The 3^rd^ centile for EFW at 30 weeks' gestation is therefore: C_0.03_(30) = exp(7.008552) = 1106 g.

The actual values for the 3^rd^, 10^th^, 50^th^, 90^th^ and 97^th^ centiles according to gestational age are presented in Table S1.

## DISCUSSION

The INTERGROWTH‐21^st^ Project provides standards for early human growth based on populations that conform to the prescriptive approach recommended by the WHO^21^, ^51^. By prescriptive, we mean that we observed a cohort of prospectively enrolled women whose risk of adverse maternal and perinatal outcomes (including fetal growth restriction) was low, based on their individual clinical profiles and the socioeconomic and demographic characteristics of the underlying geographically diverse populations. In fact, the INTERGROWTH‐21^st^ Project is unique because it has produced, for the first time, fetal ultrasound, newborn size and preterm postnatal growth datasets that have all been collected from the same underlying populations using the same rigorously applied methodologies.

We now present international EFW standards to complement the existing set, along with a formula for EFW based on HC and AC. Compared with several previous formulae^24^, we found that FL did not improve the EFW, which agrees with previous work, in particular in growth‐restricted fetuses^52^. Furthermore, it is likely that incorporating FL into the formula would increase the prediction error, as its measurement is associated with the highest inter‐ and intraobserver variability compared with AC and HC^53^.

Unusually, we lowered the starting gestational age to 22 weeks, 2 weeks below the customary cut‐off of 24 weeks' gestation for viability, for two reasons: to facilitate early recognition of fetal growth restriction around the recommended time of the second‐trimester anatomy scan and to anticipate a possible extension of the limit of viability^54^, ^55^.

At the upper end of gestation, the centiles closely match those of the INTERGROWTH‐21^st^ Newborn Size Standards at 40 weeks^7^. The 3^rd^, 50^th^ and 97^th^ EFW centiles at 40 weeks are 2554 g, 3338 g and 4121 g, respectively (Table S1), whereas for newborns (sexes combined) they are 2591 g, 3321 g and 4154 g, respectively (Figure S4). These similarities between fetal‐ and birth‐weight centiles suggest that our model is valid for developing a formula for EFW using ultrasound biometry.

**Figure 3:**
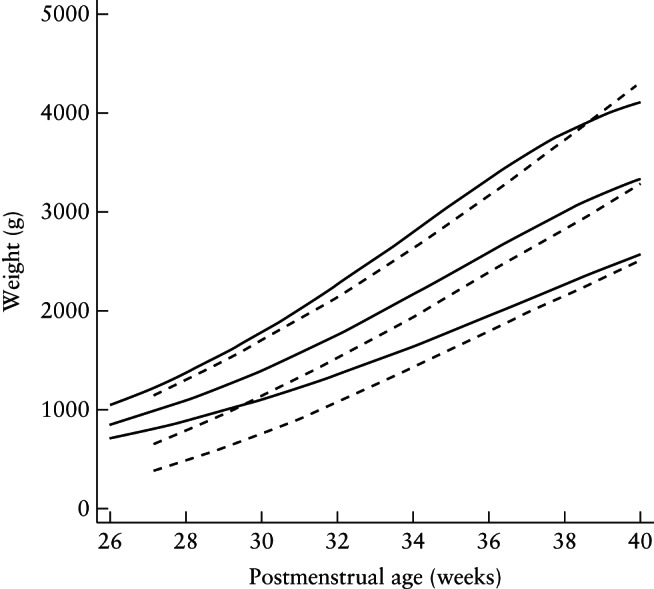
Comparison of fitted 3^rd^, 50^th^ and 97^th^ centiles for estimated fetal weight (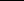) with those of INTERGROWTH‐21^st^ preterm postnatal weight, with both sexes combined (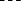).

In contrast, there are significant discrepancies earlier in pregnancy (Figure 3). For example, at 33 weeks' gestation, the 3^rd^, 50^th^ and 97^th^ EFW centiles are 1495 g, 1954 g and 2529 g, respectively (Table S1); for newborns (sexes combined), they are 1190 g, 1903 g and 2715 g, respectively. It is possible that these differences are due to an overrepresentation of small, as well as, to a lesser extent, large babies in preterm births, even in the selected pregnant and newborn populations we studied.

The EFW formula and standards we present are also unique because we avoided the many common limitations identified by previous reviews^5^, ^24^: retrospective design; use of routinely obtained measurements; suboptimal pregnancy dating strategies; variable time‐to‐birth without controlling for bias; absence of prospective ultrasound quality control, standardization and calibration of equipment; hospital‐based sampling; absence of sampling from a healthy, well‐nourished, underlying population; and no blinding of measurements.

Conversely, our standards are prescriptive, whereas reference charts describe only fetal size at a given place and time. The standards were derived prospectively, population‐based and multinational. We have shown (using several analytical strategies) that the eight populations were consistently similar and could be pooled to create international standards^51^. Uniform research methods, protocols, processes and measurement tools were used throughout; these were combined with standardized identical equipment, training, a centralized electronic data management system and close monitoring of staff. The analytical approach aimed at identifying and correcting potential biases, and followed WHO recommendations to present the observed and smoothed data and explore the best fitting model with an *a‐priori* strategy^56^.

Using ultrasound, we examined separately HC, AC and FL, providing a comprehensive evaluation of structures that have different growth patterns; these measurements are often combined to calculate EFW. There are advantages in using a summary approximation: it is the most commonly measured marker of size at birth; as birth weight is associated with morbidity and mortality, it is helpful when counseling parents and enables pediatricians to make management decisions^57^; it may also help to refine the management of large babies.

However, there are also disadvantages in using only a single summary measure of size: first, there is a loss of the most granular information available when using the individual measurements, in terms of fetal skeletal and fat‐based growth. Second, the fact that the individual measurement errors are compounded means that estimation is prone to inaccuracy; previous studies have shown that 95% prediction intervals for random error are in the region of ± 14% of birth weight, and this is a particular problem in low‐ and high‐birth‐weight babies^24^. Finally, as for other ultrasound measurements, there are numerous locally‐derived EFW equations and reference charts^24^ but, until now, no international standards existed, unlike the situation for newborn size and infant growth^7^, ^8^, ^10^. This may be, at least partly, responsible for the poor efficiency of screening strategies using biometry and EFW^58^.

Therefore, we strongly recommend that, for clinical use, all individual fetal measurements, together with the summary measure of EFW, should be used together to make clinical decisions. In perinatal medicine, there is no room for a quick, minimalist approach that might lead to the early delivery of an at‐risk fetus. Finally, implementation of the standards may raise concerns regarding the generalizability of data originating from a limited number of sites and/or a highly selected, low‐risk population. As we have argued previously^11^, having separate standards for a given country, institution or ethnic group has no biological basis and makes little sense in modern, multicultural societies. The international INTERGROWTH‐21^st^ standards describe optimal growth and can be used to assess both individuals and populations.

## Supporting information


**Appendix S1** Members of the International Fetal and Newborn Growth Consortium for the 21^st^ Century (INTERGROWTH‐21^st^ and INTERBIO‐21^st^) and its Committees

**Figure S1** Relationship between fetal weight and abdominal circumference in the final model, plotted for a fixed head circumference of 26 cm.

**Figure S2** Relationship between fetal weight and head circumference in the final model, plotted for a fixed abdominal circumference of 23 cm.

**Figure S3** Bias in estimation of fetal weight as a function of time to birth, showing mean percent prediction error and 95% CI according to time between last ultrasound scan and birth.

**Figure S4** Gestational age‐specific centiles for estimated fetal weight (blue) and birth weight (red). 3^rd^, 10^th^, 50^th^, 90^th^ and 97^th^ centiles are shown.

**Table S1** Estimated fetal weight per completed week of gestation at 3^rd^, 10^th^, 50^th^, 90^th^ and 97^th^ centiles
