# Supplementary material for: International estimated fetal weight standards of the INTERGROWTH‐21st Project
Source: Ultrasound Obstet Gynecol. 2017 Mar 5;49(4):478–86. doi: 10.1002/uog.17347 (PMC5516164; doi:10.1002/uog.17347)
Supplement: Supplementary file 1 — Appendix S1 Members of the International Fetal and Newborn Growth Consortium for the 21st Century (INTERGROWTH‐21st and INTERBIO‐21st) and its Committees [file UOG-49-478-s005.docx]

**Appendix S1**

**Members of the International Fetal and Newborn Growth Consortium for the 21^st^ Century (INTERGROWTH-21^st^ and INTERBIO-21^st^) and its Committees**

**Scientific Advisory Committee**

M Katz (Chair from January 2011), MK Bhan, C Garza, S Zaidi, A Langer, PM Rothwell (from February 2011), Sir D Weatherall (Chair until December 2010).

**Steering Committees**

INTERGROWTH-21^st^: ZA Bhutta (Chair), J Villar (Principal Investigator), S Kennedy (Project Director), DG Altman, FC Barros, E Bertino, F Burton, M Carvalho, L Cheikh Ismail, WC Chumlea, MG Gravett, YA Jaffer, A Lambert, P Lumbiganon, JA Noble, RY Pang, AT Papageorghiou, M Purwar, J Rivera, C Victora.

INTERBIO-21^st^ : R Uauy (Chair), S Kennedy (Co-Principal Investigator), J Villar (Co-Principal Investigator), DG Altman, FC Barros, J Berkley, F Burton, M Carvalho, L Cheikh Ismail, WC Chumlea, A Lambert, S Munim, S Norris, F Nosten, AT Papageorghiou, C Victora.

**Executive Committee**

J Villar (Chair), DG Altman, L Cheikh Ismail, S Kennedy, A Lambert, JA Noble, AT Papageorghiou, ZA Bhutta. In addition for INTERBIO 21^st^: R Craik, R Uauy

**Project Coordinating Unit**

J Villar (Head), L Cheikh Ismail, S Kennedy, A Lambert, AT Papageorghiou, M Shorten, L Hoch (until May 2011), HE Knight (until August 2011), EO Ohuma (from September 2010), C Cosgrove (from July 2011), I Blakey (from March 2011), S Ash (from August 2011), R Craik (from June 2011).

**Data Analysis Group**

DG Altman (Head), EO Ohuma, E Staines Urias (from April 2016), J Villar.

**Data Management Group**

DG Altman (Head), F Roseman, N Kunnawar, SH Gu, JH Wang, MH Wu, M Domingues, P Gilli, L Juodvirsiene, L Hoch (until May 2011), N Musee (until June 2011), H Al-Jabri (until October 2010), S Waller (until June 2011), C Cosgrove (from July 2011), D Muninzwa (from October 2011), EO Ohuma (from September 2010), D Yellappan (from November 2010), A Carter (from July 2011), D Reade (from June 2012), R Miller (from June 2012). In addition for INTERBIO 21^st^: I Ahmed, S Ash, C Condon, M Mainwaring, D Muninzwa, MF da Silveira, E Staines Urias, L Walusuna, S Wiladphaingern.

**Ultrasound Group**

AT Papageorghiou (Head), L Salomon (Senior external advisor), A Leston, A Mitidieri, F Al-Aamri, W Paulsene, J Sande, WKS Al-Zadjali, C Batiuk, S Bornemeier, M Carvalho, M Dighe, P Gaglioti, N Jacinta, S Jaiswal, JA Noble, K Oas, M Oberto, E Olearo, MG Owende, J Shah, S Sohoni, T Todros, M Venkataraman, S Vinayak, L Wang, D Wilson, QQ Wu, S Zaidi, Y Zhang, P Chamberlain (until September 2012), D Danelon (until July 2010), I Sarris (until June 2010), J Dhami (until July 2011), C Ioannou (until February 2012), CL Knight (from October 2010), R Napolitano (from July 2011), S Wanyonyi (from May 2012), C Pace (from January 2011), V Mkrtychyan (from June 2012). In addition for INTERBIO-21^st^: M Buckle, N Jackson, A Mitidieri, S Munim, H Mwangudzah, T Norris, J Shah, G Zainab.

**Anthropometry Group**

L Cheikh Ismail (Head), WC Chumlea (Senior external advisor), F Al-Habsi, ZA Bhutta, A Carter, M Alija, JM Jimenez-Bustos, J Kizidio, F Puglia, N Kunnawar, H Liu, S Lloyd, D Mota, R Ochieng, C Rossi, M Sanchez Luna, YJ Shen, HE Knight (until August 2011), DA Rocco (from June 2012), IO Frederick (from June 2012). In addition for INTERBIO-21^st^: J Kizidio, B Monyepote, M Salim, R Salam, VI Carrara.

**Laboratory Processing Group**

R Craik (Head), D Alam, Y Guman, J Kilonzo, A Min, V Ngami, I Olivera, G Deutsch

**Neonatal Group**

ZA Bhutta (Head), E Albernaz, M Batra, BA Bhat, E Bertino, P Di Nicola, F Giuliani, I Rovelli, K McCormick, R Ochieng, RY Pang, V Paul, V Rajan, A Wilkinson, R Uauy, A Varalda (from September 2012),

**Environmental Health Group**

B Eskenazi (Head), A Bradman, O Burnham, LA Corra, H Dolk, F Farhi, D Finkton, J Golding, A Matijasevich, T de Wet, J Villar, JJ Zhang

**Neurodevelopment Group**

A Stein (Head), M Fernandes (Coordinator), A Abubakar, J Acedo, L Aranzeta, L Cheikh Ismail, F Giuliani, D Ibanez, S Kennedy, M Kihara, E de Leon, CR Newton, S Savini, A Soria- Frisch, J Villar, K Wulff.

**Participating countries and local investigators**

*Brazil:* FC Barros (Principal Investigator), M Domingues, S Fonseca, A Leston, A Mitidieri, D Mota, IK Sclowitz, MF da Silveira.

*China:* RY Pang (Principal Investigator), YP He, Y Pan, YJ Shen, MH Wu, QQ Wu, JH Wang, Y Yuan, Y Zhang.

*India:* M Purwar (Principal Investigator), A Choudhary, S Choudhary, S Deshmukh, D Dongaonkar, M Ketkar, V Khedikar, N Kunnawar, C Mahorkar, I Mulik, K Saboo, C Shembekar, A Singh, V Taori, K Tayade, A Somani.

*Italy:* E Bertino (Principal Investigator), P Di Nicola, M Frigerio, G Gilli, P Gilli, M Giolito, F Giuliani, M Oberto, L Occhi, C Rossi, I Rovelli, F Signorile, T Todros.

*Kenya (Nairobi):* W Stones and M Carvalho (Co- Principal Investigators), J Kizidio, R Ochieng, J Shah, S Vinayak, N Musee (until June 2011), C Kisiang’ani (until July 2011), D Muninzwa (from August 2011) In addition for INTERBIO-21^st^: J Kilonzo, J Sande.

*Kenya (Kilifi)*: J Berkley (Principal Investigator), B Kemp, H Barsosio, S Mwakio, H Mwangudzah, V Ngami, M Salim, A Seale, L Walusuna.

*Oman:* YA Jaffer (Principal Investigator), J Al-Abri, J Al-Abduwani, FM Al-Habsi, H Al-Lawatiya, B Al-Rashidiya, WKS Al-Zadjali, FR Juangco, M Venkataraman, H Al-Jabri (until October 2010), D Yellappan (from November 2010).

*Pakistan*: S Munim and G Zainab (Co-Principal Investigators), I Ahmed, D Alam, A Raza, R Salam.

*South Africa*: S Norris (Principal Investigator), Y Guman, T Lephoto, S Macauley, L Malgas.

*Thailand*: F Nosten (Principal Investigator), N Jackson, R McGready, A Min, VI Cararra, S Wiladphaingern.

*UK:* S Kennedy (Principal Investigator), L Cheikh Ismail, A Lambert, S Lloyd, R Napolitano (from July 2011), EO Ohuma, AT Papageorghiou, B Patel, F Puglia, F Roseman, S Roseman, C Ioannou (until February 2012), I Sarris (until June 2010). In addition for INTERBIO-21^st^: S Ash, M Baricco, A Capp, R Craik, S Hussein, A Laister, T Lewis, E Maggiora, T Norris, M Sharps, A Varalda, R Carew.

*USA:* MG Gravett (Principal Investigator), C Batiuk, M Batra, S Bornemeier, M Dighe, K Oas, W Paulsene, D Wilson, IO Frederick, HF Andersen, SE Abbott, AA Carter, H Algren, DA Rocco, TK Sorensen, D Enquobahrie, S Waller (until June 2011).
