# Supplementary figures and images for: International estimated fetal weight standards of the INTERGROWTH‐21st Project
Source: Ultrasound Obstet Gynecol. 2017 Mar 5;49(4):478–86. doi: 10.1002/uog.17347 (PMC5516164; doi:10.1002/uog.17347)

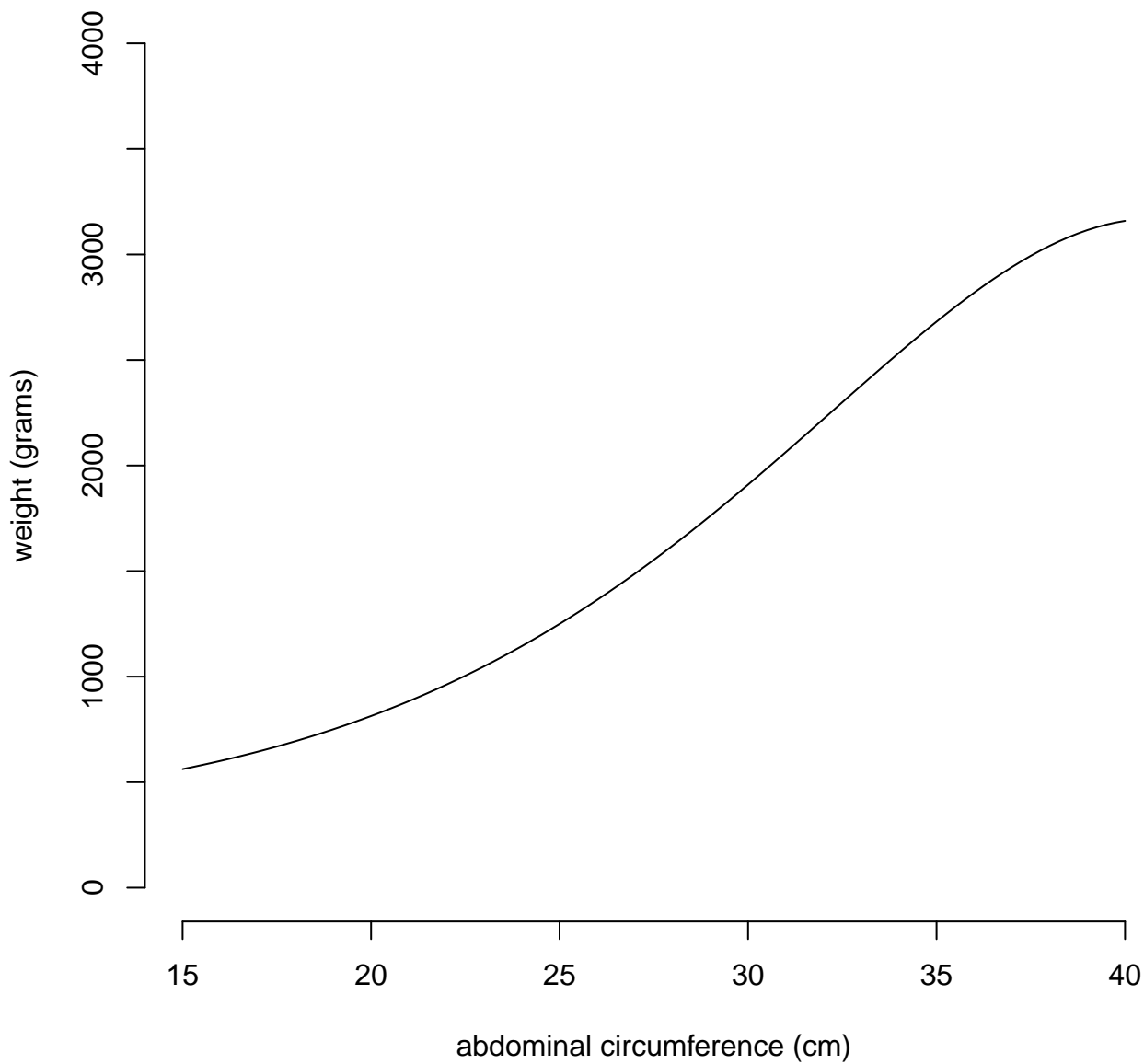

Supplement: Supplementary file 2 — Figure S1 Relationship between fetal weight and abdominal circumference in the final model, plotted for a fixed head circumference of 26 cm. [file UOG-49-478-s004.pdf]

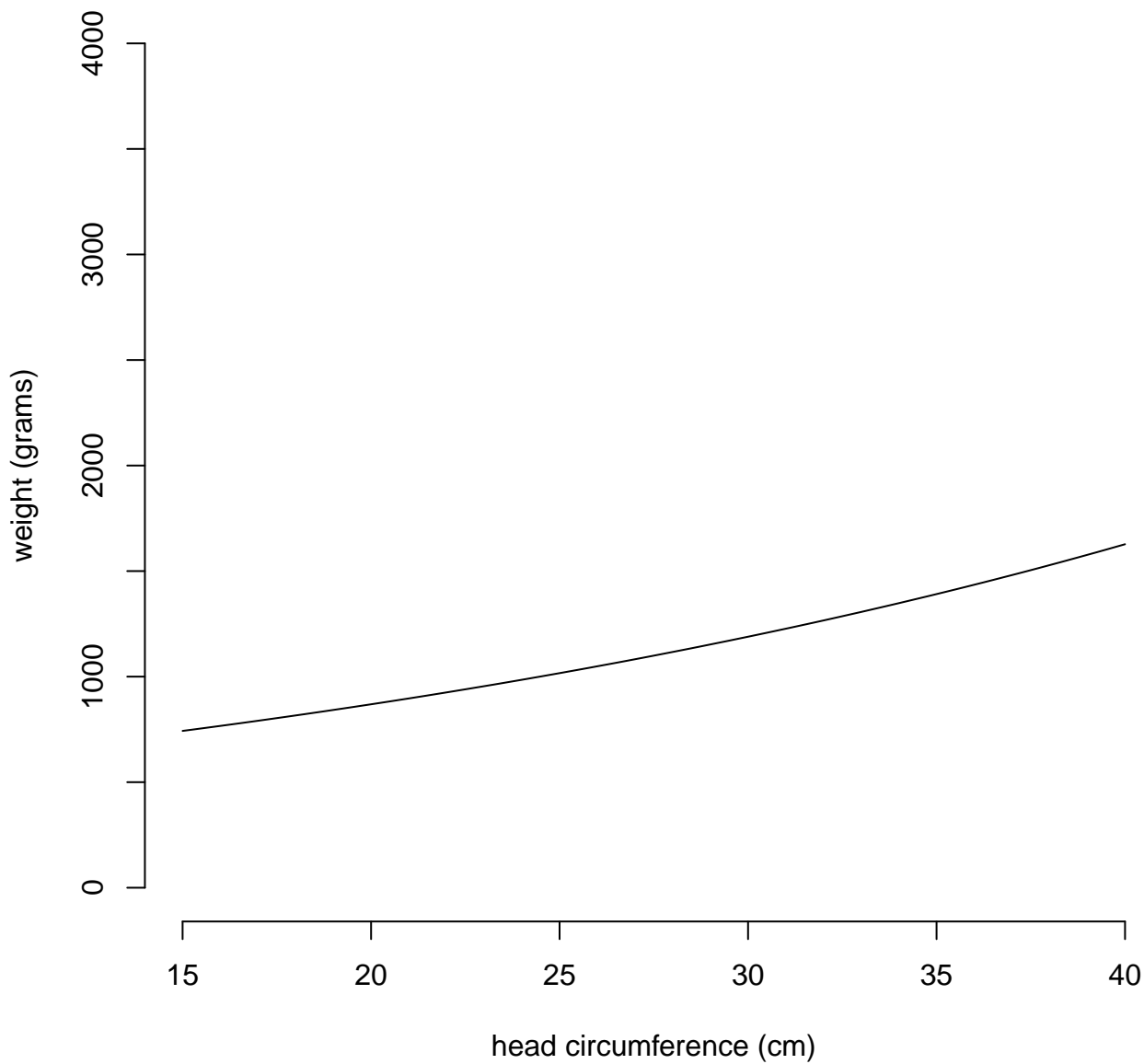

Supplement: Supplementary file 3 — Figure S2 Relationship between fetal weight and head circumference in the final model, plotted for a fixed abdominal circumference of 23 cm. [file UOG-49-478-s003.pdf]

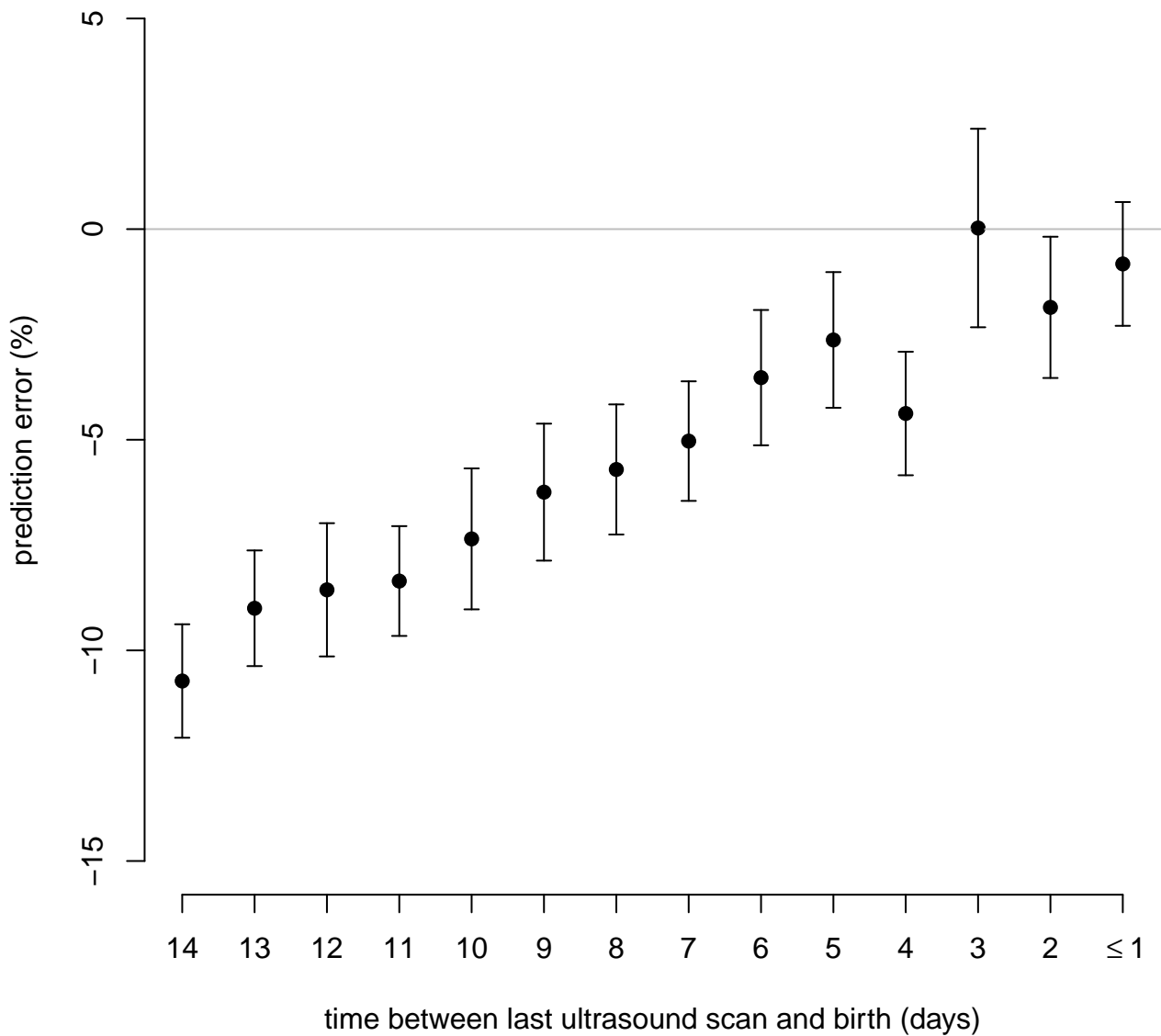

Supplement: Supplementary file 4 — Figure S3 Bias in estimation of fetal weight as a function of time to birth, showing mean percent prediction error and 95% CI according to time between last ultrasound scan and birth. [file UOG-49-478-s002.pdf]

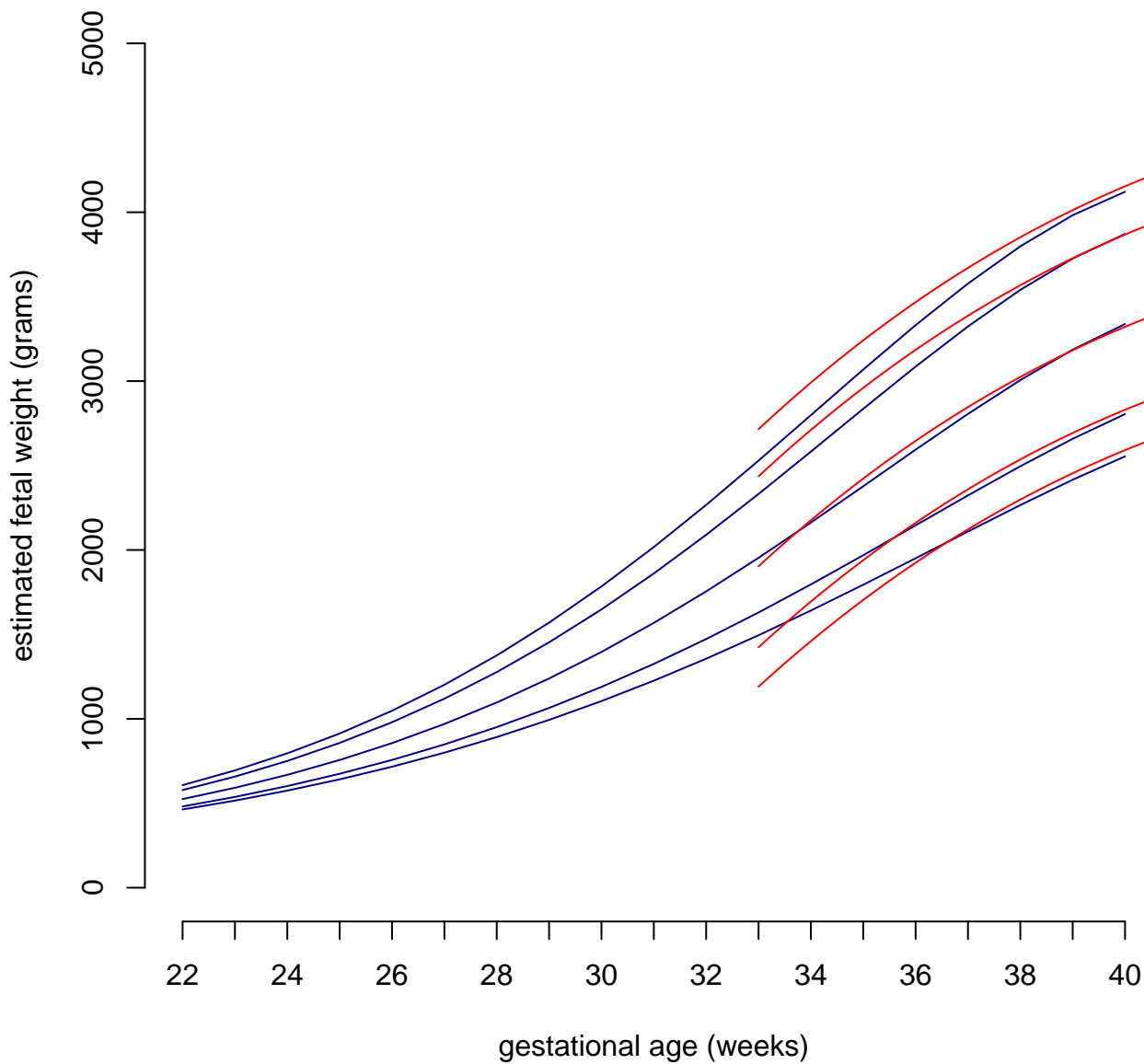

Supplement: Supplementary file 5 — Figure S4 Gestational age‐specific centiles for estimated fetal weight (blue) and birth weight (red). 3rd, 10th, 50th, 90th and 97th centiles are shown. [file UOG-49-478-s001.pdf]
