# Supplementary material for: International estimated fetal weight standards of the INTERGROWTH‐21st Project
Source: Ultrasound Obstet Gynecol. 2017 Mar 5;49(4):478–86. doi: 10.1002/uog.17347 (PMC5516164; doi:10.1002/uog.17347)
Supplement: Supplementary file 6 — Table S1 Estimated fetal weight per completed week of gestation at 3rd, 10th, 50th, 90th and 97th centiles [file UOG-49-478-s006.docx]

**Table S1** Estimated fetal weight per completed week of gestation at 3^rd^, 10^th^, 50^th^, 90^th^ and 97^th^ centile

|  | EFW (g) | | | | |
| --- | --- | --- | --- | --- | --- |
| GA  (weeks) | 3^rd^ centile | 10^th^ centile | 50^th^ centile | 90^th^ centile | 97^th^ centile |
| 22 | 463 | 481 | 525 | 578 | 607 |
| 23 | 516 | 538 | 592 | 658 | 695 |
| 24 | 575 | 602 | 669 | 751 | 796 |
| 25 | 641 | 674 | 756 | 858 | 913 |
| 26 | 716 | 757 | 856 | 980 | 1048 |
| 27 | 800 | 849 | 969 | 1119 | 1202 |
| 28 | 892 | 951 | 1097 | 1276 | 1375 |
| 29 | 994 | 1065 | 1239 | 1452 | 1569 |
| 30 | 1106 | 1190 | 1396 | 1647 | 1783 |
| 31 | 1227 | 1326 | 1568 | 1860 | 2016 |
| 32 | 1357 | 1473 | 1755 | 2089 | 2266 |
| 33 | 1495 | 1630 | 1954 | 2332 | 2529 |
| 34 | 1641 | 1795 | 2162 | 2583 | 2800 |
| 35 | 1792 | 1967 | 2378 | 2838 | 3071 |
| 36 | 1948 | 2144 | 2594 | 3089 | 3335 |
| 37 | 2106 | 2321 | 2806 | 3326 | 3582 |
| 38 | 2265 | 2495 | 3006 | 3541 | 3799 |
| 39 | 2422 | 2663 | 3186 | 3722 | 3976 |
| 40 | 2574 | 2818 | 3338 | 3858 | 4101 |
